# In vivo analysis of *Saccharomyces cerevisiae* plasma membrane ATPase Pma1p isoforms with increased in vitro H^+^/ATP stoichiometry

**DOI:** 10.1007/s10482-012-9730-2

**Published:** 2012-04-10

**Authors:** Stefan de Kok, Duygu Yilmaz, Jean-Marc Daran, Jack T. Pronk, Antonius J. A. van Maris

**Affiliations:** Department of Biotechnology, Delft University of Technology and Kluyver Centre for Genomics of Industrial Fermentation, Julianalaan 67, 2628 BC Delft, The Netherlands

**Keywords:** Pma1, Ser800Ala, Glu803Gln, Maltose, Yeast, Proton symport

## Abstract

Plasma membrane H^+^-ATPase isoforms with increased H^+^/ATP ratios represent a desirable asset in yeast metabolic engineering. In vivo proton coupling of two previously reported Pma1p isoforms (Ser800Ala, Glu803Gln) with increased in vitro H^+^/ATP stoichiometries was analysed by measuring biomass yields of anaerobic maltose-limited chemostat cultures expressing only the different *PMA1* alleles. In vivo H^+^/ATP stoichiometries of wildtype Pma1p and the two isoforms did not differ significantly.

## Introduction

Plasma membrane H^+^-ATPases are ubiquitous enzymes that play an important role in eukaryotic physiology by using the free energy from ATP hydrolysis to pump protons from the cytosol, across the plasma membrane and out of the cell. In this way, cells maintain intracellular pH homeostasis and generate a proton motive force (PMF), which can be used to drive many crucial transport processes (Serrano 1991; Burgstaller 1997; Van Maris et al. 2004). *Saccharomyces cerevisiae* contains two distinct plasma membrane H^+^-ATPases encoded by the essential gene *PMA1* and the non-essential gene *PMA2* (Serrano et al. 1986; Schlesser et al. 1988). The well-characterised plasma membrane H^+^-ATPase of *S. cerevisiae* (Serrano 1989; Morsomme et al. 2000; Morth et al. 2010) expels one proton per ATP molecule hydrolysed (Van Leeuwen et al. 1992; Weusthuis et al. 1993), even though the Gibbs free-energy of ATP hydrolysis (around −45 kJ mol^−1^ under physiological conditions (Canelas et al. 2011)) should be sufficient to drive export of 2 protons (+19 kJ mol protons^−1^ at a PMF of −200 mV (Serrano 1991)). The H^+^/ATP stoichiometry of the plasma membrane H^+^-ATPase determines the ATP requirement for cellular homeostasis and maintenance of the PMF. Moreover, it influences the biomass yield on substrates whose import makes use of the PMF (e.g., maltose and NH_4_^+^) (Van Leeuwen et al. 1992; Weusthuis et al. 1993; Marini et al. 1997), it can influence tolerance to both low pH and weak organic acids (Verduyn et al. 1992; Piper et al. 1998; Abbott et al. 2007) and it can have a crucial impact on the stoichiometry and kinetics of organic acid production by engineered strains of *S. cerevisiae* (Van Maris et al. 2004; Sauer et al. 2008; Abbott et al. 2009). Increasing the H^+^/ATP stoichiometry of the *S. cerevisiae* plasma membrane H^+^-ATPase could therefore present many interesting opportunities for metabolic engineering.

Two isoforms of the *S. cerevisiae* plasma membrane H^+^-ATPase Pma1p have been described that displayed an increased in vitro H^+^/ATP stoichiometry: Pma1p^Ser800Ala^ (Guerra et al. 2007) and Pma1p^Glu803Gln^ (Petrov et al. 2000). However, no improved tolerance to low pH was observed after introducing the Glu803Gln mutation in *PMA1* (Guerra et al. 2007). The present study investigates whether the in vivo H^+^/ATP stoichiometry of *S. cerevisiae* Pma1p isoforms can be analysed via the anaerobic biomass yield on maltose of engineered strains. In *S. cerevisiae* maltose is imported via a proton-symport mechanism (Van Leeuwen et al. 1992). Due to subsequent proton export by the plasma membrane H^+^-ATPase at a stoichiometry of 1 H^+^/ATP, conversion of the disaccharide maltose to ethanol only yields 3 ATP (1.5 ATP per hexose equivalent) (Van Leeuwen et al. 1992; Weusthuis et al. 1993). As a result, the anaerobic biomass yield on maltose is 25 % lower per hexose equivalent than the anaerobic biomass yield on glucose (2 ATP per hexose) (Van Leeuwen et al. 1992; Weusthuis et al. 1993). In theory, an increased stoichiometry of the plasma membrane H^+^-ATPase will increase the biomass yield on maltose due to a decreased energy requirement of all processes that require proton extrusion (e.g., maintenance and generation of the PMF and import of maltose and NH_4_^+^). Even when only the lower ATP-requirement for maltose import is taken into account (Weusthuis et al. 1993), a stoichiometry of 2 H^+^/ATP is already expected to result in a 17 % increase of the biomass yield on maltose. To characterise the in vivo H^+^/ATP stoichiometry of the Ser800Ala and Glu803Gln isoforms of Pma1p, the wild-type *PMA1* allele was replaced by the corresponding *PMA1* mutant alleles in a *pma2*Δ background and the anaerobic biomass yields on maltose were compared to those of an isogenic *PMA1* *pma2*Δ reference strain.

## Introduction of Ser800Ala and Glu803Gln mutations in *PMA1*

To introduce the Ser800Ala (TCC → GCT) and Glu803Gln (GAA → CAA) mutations into *PMA1*, DNA from the *Kpn*I site in *PMA1* until the *Ngo*MIV site in *LEU1* was amplified from CEN.PK113-7D genomic DNA using primers PMA1 Fw and LEU1 Rv (for primers, see Table 1) and cloned into pENTR/D-TOPO using Gateway technology (Invitrogen, Carlsbad, USA), resulting in pUD109 (for plasmids, see Table 2). To introduce extra restriction sites in the intergenic region between *PMA1* and *LEU1,* DNA was amplified from pUD109 using primers LEU1p Fw and LEU1p Rv. The resulting PCR product was restricted with *Xba*I and *Hind*III and ligated into pUD109, resulting in pUD113. To introduce point mutations in *PMA1*, the *Kpn*I-*Sal*I fragment of pUD117 and pUD118, containing synthesized parts of *PMA1* including the Ser800Ala (TCC → GCT) and Glu803Gln (GAA → CAA) mutations, were ligated into pUD113, resulting in pUD119 and pUD120 (Table 2). To introduce the hygromycin B resistance marker *hphNT1*, a *Spe*I-*Bsi*WI fragment of pUG-*hphNT1* was ligated into pUD119 and pUD120, resulting in pUD124 and pUD125, respectively. The *Kpn*I-*Ngo*MIV fragment of pUD124 and pUD125 was transformed to CEN.PK113-7D resulting in IMI058 and IMI059, respectively (for strains, see Table 3). Correct integration of the cassette was confirmed via PCR using primer pairs PMA1 Ctrl Fw/hphNT1 Rv and hphNT1 Fw/LEU1 Ctrl Rv. To remove the hygromycin B resistance marker gene *hphNT1*, IMI058 and IMI059 were transformed with pSH65 and—after marker removal via the Cre/loxP system (Gueldener et al. 2002) and curing of the pSH65 plasmid—designated IMI062 and IMI063, respectively. To knockout *PMA2*, a cassette was amplified from pUG6 using primers PMA2 KO Fw and PMA2 KO Rv and transformed into CEN.PK113-7D, IMI062 and IMI063, resulting in IMK328, IMX051B and IMX052, respectively. Correct knockout was confirmed via PCR using the primer pairs PMA2 Ctrl Fw/KanMX Rv and KanMX Fw/PMA2 Ctrl Rv. Presence of the introduced point mutations was verified by duplicate amplification of *PMA1* using primers PMA1 Ctrl Fw and LEU1 Ctrl Rv and sequencing approximately 200 bp up- and downstream of the introduced mutations (Baseclear BV, Leiden, The Netherlands). Strain maintenance, yeast transformations and molecular biology techniques were performed as described previously (De Kok et al. 2011).

**Table 1:** Primers used in this study

Primer name	Sequence (5′→3′)
PMA1 Fw	CACCGGGTACCAACATTTACAACGCTG
LEU1 Rv	CAACTCTTCTGACCTTTCTGCC
LEU1p Fw	CTCTAGACACTAGTATGCCGTACGTGACTCAGTTTAGTCTGACCTTC
LEU1p Rv	CCTTCGAAAGCTTGTGGAG
PMA1 Ctrl Fw	GGATCCACCAAGAGACGATACTG
LEU1 Ctrl Rv	CCGAAATATGGAACGCCGAACTG
hphNT1 Fw	ACGCGGATTTCGGCTCCAAC
hphNT1 Rv	AGACGTCGCGGTGAGTTCAG
PMA2 KO Fw	TCGTTGCTGTGTGCTAGTACAATTTAAGCAAAAGGAAACTGTTTTGCGTTCAGCTGAAGCTTCGTACGC
PMA2 KO Rv	CTTGGTATCGACAAATTGAAATGAAAATGAGGAATAACAAAAAGGAGATCGCATAGGCCACTAGTGGATCTG
PMA2 Ctrl Fw	GGCGGTGTGATGGTACTTC
PMA2 Ctrl Rv	CGGCCTACTTCTGATATGTGG
KanMX Fw	TCGTATGTGAATGCTGGTCG
KanMX Rv	CGCACGTCAAGACTGTCAAG

**Table 2:** Plasmids used in this study

Plasmid	Characteristic	Reference/source
pENTR/D-TOPO	Gateway entry clone	Invitrogen, USA
pUG6	PCR template for *loxP*-*KanMX4*-*loxP* cassette	(Gueldener et al. 2002)
pSH65	Centromeric plasmid, *ble* ^*R*^, P_*GAL1*_-*Cre*-T_*CYC1*_	(Gueldener et al. 2002)
pUG-*hphNT1*	PCR template for *loxP*-*hphNT1*-*loxP* cassette	(De Kok et al. 2011)
pUD117	pUC57, synthetic construct ‘*PMA1* ^*S800A*^ *’*	Baseclear BV, The Netherlands
pUD118	pUC57, synthetic construct ‘*PMA1* ^*E803Q’*^	Baseclear BV, The Netherlands
pUD109	Gateway entry clone, ‘*PMA1*-*LEU1’*	This study
pUD113	Gateway entry clone, ‘*PMA1*-*multiple cloning site*-*LEU1’*	This study
pUD119	Gateway entry clone, ‘*PMA1* ^*S800A*^ -*multiple cloning site*-*LEU1’*	This study
pUD120	Gateway entry clone, ‘*PMA1* ^*E803Q*^ -*multiple cloning site*-*LEU1’*	This study
pUD124	Gateway entry clone, ‘*PMA1* ^*S800A*^ -*loxP*-*hphNT1*-*loxP*-*LEU1’*	This study
pUD125	Gateway entry clone, ‘*PMA1* ^*E803Q*^ -*loxP*-*hphNT1*-*loxP*-*LEU1’*	This study

**Table 3:** *Saccharomyces cerevisiae* strains used in this study

Strain	Relevant genotype	Reference
CEN.PK113-7D	MATa *PMA1 PMA2*	(Van Dijken et al. 2000, Entian and Kotter 2007)
IMK328	MATa *PMA1* *pma2*::*loxP*-*KanMX4*-*loxP*	This study
IMI058	MATa *PMA1* ^*S800A*^-*loxP*-*hphNT1*-*loxP PMA2*	This study
IMI059	MATa *PMA1* ^*E803Q*^-*loxP*-*hphNT1*-*loxP PMA2*	This study
IMI062	MATa *PMA1* ^*S800A*^ *PMA2*	This study
IMI063	MATa *PMA1* ^*E803Q*^ *PMA2*	This study
IMX051B	MATa *PMA1* ^*S800A*^ *pma2*::*loxP*-*KanMX4*-*loxP*	This study
IMX052	MATa *PMA1* ^*E803Q*^ *pma2*::*loxP*-*KanMX4*-*loxP*	This study

## Analysis of the in vivo stoichiometry of Pma1p^Ser800Ala^ and Pma1p^Glu803Gln^

To analyse in vivo H^+^/ATP stoichiometry of the Pma1p isoforms, anaerobic chemostat experiments with maltose as the sole carbon source were performed at pH 5.0 as described previously (De Kok et al. 2011). To prevent evolutionary adaptation, the cultures were sampled within 12 volume changes. In agreement with model predictions based on a H^+^/ATP stoichiometry of 1.0 and previous observations under the same experimental conditions, the anaerobic biomass yield on maltose of the reference strain CEN.PK113-7D (*PMA1 PMA2*) was 24 ± 0 % lower per hexose equivalent than the anaerobic biomass yield on glucose (Table 4). The biomass yield of the engineered strains IMX051B (*PMA1* ^*Ser800Ala*^ *pma2*Δ) and IMX052 (*PMA1* ^*Glu803Gln*^ *pma2*Δ) was not higher than the yield of the reference strain CEN.PK113-7D (*PMA1 PMA2*) or the isogenic strain IMK328 (*PMA1 pma2*Δ) (Table 4). At the end of the chemostat experiments, genomic DNA was extracted and used for duplicate amplification of part of *PMA1*. Subsequent sequencing confirmed that the introduced mutations were still present. Apparently, the Ser800Ala and Glu803Gln mutations in *PMA1* did not increase the in vivo H^+^/ATP stoichiometry under the tested conditions, in contrast to what has been reported previously using in vitro assays (Petrov et al. 2000; Guerra et al. 2007).To test whether these contradictory results were due to differences in pH used in the in vivo (pH 5.0) and in vitro (pH 6.7) experiments, the chemostat experiments were repeated at pH 6.7. Also under these conditions, the difference in anaerobic biomass yield on glucose and maltose of the reference strain CEN.PK113-7D (*PMA1 PMA2*) at pH 6.7 was 24 ± 0 % (Table 4). Interestingly, at pH 6.7 deletion of *PMA2* seemed to increase the biomass yield on maltose by 5.4 ± 0.0 % when comparing the reference strain CEN.PK113-7D (*PMA1 PMA2*) and IMK328 (*PMA1 pma2*Δ). However, the biomass yields on maltose of the engineered strains IMX051B (*PMA1* ^*Ser800Ala*^ *pma2*Δ) and IMX052 (*PMA1* ^*Glu803Gln*^ *pma2*Δ) were identical to the isogenic reference strain IMK328 (*PMA1 pma2*Δ) (Table 4). Thus, at both pH 5.0 and pH 6.7 introduction of the Ser800Ala and Glu803Gln isoforms in Pma1p did not increase the in vivo H^+^/ATP stoichiometry.

**Table 4:** Anaerobic biomass yields of *Saccharomyces cerevisiae* strains CEN.PK113-7D (*PMA1 PMA2*), IMK328 (*PMA1 pma2*Δ), IMX051B (*PMA1* ^*Ser800Ala*^ *pma2*Δ) and IMX052 (*PMA1* ^*Glu803Gln*^ *pma2*Δ) in anaerobic sugar-limited chemostat cultures at a dilution rate of 0.10 h^−1^. Averages and mean deviations were obtained from duplicate cultures

Strain	Relevant genotype	Carbon source	Biomass yield (g g gluc eq^−1^)	
			pH 5.0	pH 6.7
CEN.PK113-7D	*PMA1 PMA2*	Glucose	0.095 ± 0.002	0.087 ± 0.001
CEN.PK113-7D	*PMA1 PMA2*	Maltose	0.072 ± 0.000	0.066 ± 0.000
IMK328	*PMA1 pma2*Δ	Maltose	0.072 ± 0.001	0.070 ± 0.000
IMX051B	*PMA1* ^*Ser800Ala*^ *pma2*Δ	Maltose	0.073 ± 0.001	0.069 ± 0.002
IMX052	*PMA1* ^*Glu803Gln*^ *pma2*Δ	Maltose	0.073 ± 0.001	0.069 ± 0.001

In vitro studies are an essential tool in gaining increased understanding of membrane proteins such as H^+^-ATPase (Serrano 1989; Morsomme et al. 2000; Morth et al. 2010). Several factors may explain why the Ser800Ala and Glu803Gln isoforms of Pma1p H^+^-ATPase isoforms, which were clearly shown to translocate 2–3 protons per ATP in vitro (Petrov et al. 2000; Guerra et al. 2007), did not lead to a significantly increased in vivo H^+^/ATP stoichiometry in the anaerobic, maltose-limited cultures. Even when in vitro studies attempt to mimic in vivo conditions (e.g., pH and osmolarity), subtle differences in membrane composition between the plasma membrane and secretory vesicle membrane (Van der Rest et al. 1995) might affect the three-dimensional structure and functioning of the plasma membrane H^+^-ATPase. Additionally, thermodynamics of the proton-motive force and/or ATP hydrolysis may be different under in vitro and in vivo conditions. If the PMF in the secretory vesicles, which has not been measured (Petrov et al. 2000; Guerra et al. 2007), is significantly lower than the in vivo PMF, this would make an increased H^+^/ATP stoichiometry thermodynamically easier to achieve in vitro, but not in vivo. This difference between the in vitro and in vivo thermodynamic potential of the H^+^-ATPase becomes even more striking for the free energy of ATP hydrolysis. In the in vitro assays, ADP and inorganic phosphate were not added to the reaction mixture and only ATP was added from the start. Especially during the early stages of the assay, which coincides with the determination of the H^+^/ATP stoichiometry, this created a non-physiologically high driving force for ATP hydrolysis, which will drastically exceed the estimated −45 kJ mol^−1^ under physiological conditions (Canelas et al. 2011). Analogously, due to cellular homeostasis and flux-versus-stoichiometry constraints techniques such as membrane potential determination or extracellular acidification measurements do not allow accurate in vivo analysis of the H^+^/ATP stoichiometry. Therefore, the method presented in this study, in which in vivo proton coupling of plasma membrane H^+^-ATPase isoforms was analysed via its impact on the biomass yields of anaerobic, maltose-grown cultures, provides a useful tool in the continuing search for Pma1p isoforms and/or heterologous plasma membrane H^+^-ATPases with an in vivo H^+^/ATP ratio above 1.0 in growing yeast cultures.
